# Early Odontogenic Differentiation of Dental Pulp Stem Cells Treated with Nanohydroxyapatite–Silica–Glass Ionomer Cement

**DOI:** 10.3390/polym12092125

**Published:** 2020-09-17

**Authors:** Hii Siew Ching, Kannan Thirumulu Ponnuraj, Norhayati Luddin, Ismail Ab Rahman, Nik Rozainah Nik Abdul Ghani

**Affiliations:** 1 School of Dental Sciences, Universiti Sains Malaysia, Kubang Kerian 16150, Kelantan, Malaysia; siewching_hii@yahoo.com (H.S.C.); norhayatikck@usm.my (N.L.); arismail@usm.my (I.A.R.); rozainah@usm.my (N.R.N.A.G.); 2 Human Genome Centre, School of Medical Sciences, Universiti Sains Malaysia, Kubang Kerian 16150, Kelantan, Malaysia

**Keywords:** cell differentiation, stem cells, gene expression, odontogenesis, dental material, nanohydroxyapatite–silica–glass ionomer cement

## Abstract

This study aimed to investigate the effects of nanohydroxyapatite–silica–glass ionomer cement (nanoHA–silica–GIC) on the differentiation of dental pulp stem cells (DPSCs) into odontogenic lineage. DPSCs were cultured in complete Minimum Essential Medium Eagle—Alpha Modification (*α*-MEM) with or without nanoHA–silica–GIC extract and conventional glass ionomer cement (cGIC) extract. Odontogenic differentiation of DPSCs was evaluated by real-time reverse transcription polymerase chain reaction (rRT–PCR) for odontogenic markers: dentin sialophosphoprotein (*DSPP*), dentin matrix protein 1 (*DMP1*), osteocalcin (*OCN*), osteopontin (*OPN*), alkaline phosphatase (*ALP*), collagen type I (*COL1A1*), and runt-related transcription factor 2 (*RUNX2*) on day 1, 7, 10, 14, and 21, which were normalized to the house keeping gene glyceraldehyde-3-phosphate dehydrogenase (*GAPDH*). Untreated DPSCs were used as a control throughout the study. The expressions of *DSPP* and *DMP1* were higher on days 7 and 10, that of *OCN* on day 10, those of *OPN* and *ALP* on day 14, and that of *RUNX2* on day 1; *COL1A1* exhibited a time-dependent increase from day 7 to day 14. Despite the above time-dependent variations, the expressions were comparable at a concentration of 6.25 mg/mL between the nanoHA–silica–GIC and cGIC groups. This offers empirical support that nanoHA–silica–GIC plays a role in the odontogenic differentiation of DPSCs.

## 1. Introduction

Dental pulp stem cells (DPSCs) were first isolated by Gronthos and his colleagues from human dental pulp [1]. DPSCs can be used directly for dental therapy as these cells have the ability to differentiate into odontoblasts. Besides that, they have also been used as an in vitro model to evaluate newly developed bioactive materials [2]. The use of glass ionomer cements (GICs) was reported by Wilson and Kent in 1970s [3]. GICs are widely used in dental application due to their many advantages such as biocompatibility, long-term release of fluoride which acts as an anticariogenic agent, elasticity similar to dentin, and ability to bond to the tooth structure directly [4,5]. Despite their advantages, they have certain limitations such as susceptibility to dehydration and poor physical and mechanical properties [6], which have limited the extensive use of GICs as a filling material in dentistry. In order to overcome the poor mechanical properties of GICs, a number of modifications of conventional GICs (cGICs) have been done such as incorporation of fiber-reinforcement, hydroxyapatite, and zirconia into GICs [7,8]. GIC is composed of two main ingredients required for maintaining its desirable properties, namely, polymeric water-soluble acid and ion-leachable glass. For this purpose, aluminosilicate glass is used to prepare the GIC powder that provides a constant source of metal ions for the cement-forming reaction [9]. Glasses used in the GIC are complex and have three major components: silica (SiO_2_), alumina (Al_2_O_3_), and calcium fluoride (CaF_2_). In addition, they also contain sodium fluoride (NaF) and cryolite (Na_3_AlF_6_) or aluminium phosphate (AlPO_4_) [10,11]. However, alumina and silica are the two main components of GIC powder that form the “backbone and skeletal structure of the glass” [10]. The second component of GIC is the liquid containing polyacids known as polyalkenoics. Since the early formulations of GIC comprised about 40–50% of aqueous solution of acrylic acid [9,12] and had few disadvantages such as high viscosity and a short shelf life, acrylic acid was later co-polymerized with various homopolymers or copolymers of carboxylic acids such as acrylic acid, maleic acid, itaconic acid, and tricarboxylic acid. Thus, glass ionomers are complex materials with a predominantly silica gel-like matrix as a result of the reaction between an aqueous poly acrylic acid solution and a fluoro–alumino–silicate glass powder. The partially dissolved remnant glass cores act as fillers within the matrix which is composed of poly salt bridges and polymer chains [13]. Barry et al. [14] also suggested that a set matrix of GIC is a highly intricate network of aluminium and calcium polyacrylate gel which contains ample fluoride inside. Nicholson in 2010 also reported that three regions can be identified in the structure of GIC which include a core of glass particles surrounded by a layer of silica and lastly the matrix of the cement [15].

Hydroxyapatite has excellent biocompatibility and can promote osteoconduction and osteointegration. It is preferred as the biomaterial of choice in both dentistry and orthopedics [16,17]. Due to the development in nanotechnology, nano-hydroxyapatite (nanoHA) has found a place in dental applications [18]. Besides that, nanoHA has been used as an additive material with the aim of improving the already existing dental materials in restorative dentistry [19]. Nano-hydroxyapatite–silica (nanoHA–silica) has been synthesized by a one-pot sol-gel technique [20,21]. The nanoHA–silica alone was demonstrated to be non-genotoxic based on a comet assay [22]. The nanoHA–silica consists of a mixture of spherical silica particles (~50 nm) and rod-shaped HA particles ranging between 100–200 nm; moreover, the incorporation of nanoHA–silica into cGIC resulted in better shear bond strength and mechanical properties (compressive and flexural strengths and Vickers hardness) [20,21,23]. Moheet and colleagues, based on their several characterization studies on nanoHA–silica powder and nanohydroxyapatite-silica-glass ionomer cement (nanoHA–silica–GIC) composite, concluded that the nanopowder was successfully incorporated into the cGIC based on the elemental peaks and molecular interactions [23]. It was also suggested that the homogenous particle distribution might have contributed to the enhancement of mechanical properties of the modified cement resulting in the enhancement of GIC cement matrix [20]. Moreover, the high degree of cross linking between silica and GIC makes the nanoHA–silica–GIC much stronger in hardness compared to cGIC [21]. Studies have been previously carried out to investigate the odontogenic differentiation potential of human dental pulp cells (hDPCs) from deciduous teeth using pre-reacted glass–ionomer cement [24], hydrogel scaffolds from decellularized bone extracellular matrix and collagen type 1 [25], dental pulp stem cells (DPSCs) on tricalcium phosphate (TCP) scaffolds [26], and three bioactive materials, namely, nanoHA, mineral trioxide aggregate, and calcium-enriched mixture cements [27]. Kwon and colleagues investigated the effects of triethylene glycol dimethacrylate (TEGDMA) and 2-hydroxyethyl methacrylate (HEMA) on the odontogenic differentiation of human dental pulp cells (HDPCs) [28]. However, Bakapoulou et al. reported that HEMA exhibited a cytotoxic effect on dental pulp cells that can disturb the odontogenic differentiation potential of HEMA which could lead to compromising pulp-tissue homeostasis and repair [29]. Thus, there has always been a quest to explore the odontogenic differentiation potential of materials in dentistry. However, there is still a dearth of information on their odontogenic potential in dental stem cells. Bearing in mind the above properties, the present study was principally aimed at evaluating the effect of nanoHA–silica–GIC on the differentiation of DPSCs into odontogenic lineage.

## 2. Materials and Methods

### 2.1. Cell Culture

DPSCs (AllCells, Alameda, CA, USA; Cat no. DP003F) were grown in Minimum Essential Medium Eagle—Alpha Modification (α-MEM, Gibco, Grand Island, NY, USA) supplemented with 10% fetal bovine serum (Gibco, Grand Island, NY, USA) and 1% of penicillin/streptomycin (100 U/mL penicillin and 100 g/mL streptomycin, Gibco, Grand Island, NY, USA) and were incubated at 37 °C in a 5% CO_2_ incubator until 70–80% confluence. The DPSCs were revived from cryopreservation and sub-cultured twice before seeding for treatment. A negative control group (DPSCs without treatment) was also included in this study. Both groups of DPSCs, one with nanoHA–silica–GIC and the other with cGIC, were incubated and harvested at different incubation times (day 1, 7, 10, 14, and 21). Passage 7 was used for the current study.

### 2.2. Material Preparation

NanoHA–silica–GIC and commercial cGIC Fuji IX GP (GC Corporation, Tokyo, Japan) were used in the current study. NanoHA–silica–GIC was prepared by the addition of nanoHA–silica to cGIC. cGIC was prepared according to the instructions provided by the manufacturer. Synthesis of nanoHA–silica powder was carried out as previously described [20]: 100 mg of nanoHA–silica powder was weighed and added to 1900 mg of cGIC powder to obtain a 5% nanoHA–silica–GIC powder mixture. This 5% nanoHA–silica–GIC powder mixture was ground manually using a pestle and mortar. The Fuji XI liquid was added to the powder mixture based on the manufacturer’s recommended ratio of 1:1 (powder: liquid), which was 0.36 g of powder to 0.10 g of liquid, and mixed using an agate spatula. The cement was then placed into a 10 mm × 2 mm mould and left for setting.

In the meantime, cGIC was prepared by spatulation of the powder into the Fuji XI liquid at a ratio of 1:1 (0.36 g of powder to 0.10 g of liquid) and mixed. This cement was also introduced into a 10 mm × 2 mm mould and left for setting. After 24 h of incubation, the cements were removed from the molds, weighed, and sterilized under ultraviolet radiation for 30 min. Then, the cements were introduced individually into a centrifuge tube with a suitable amount of complete growth medium (standardized at 200 mg/mL). The medium containing the materials was incubated at 37 °C with 5% CO_2_ for 72 h. After incubation, the material extracts were filtered using a 0.22 µm syringe filter into a centrifuge tube [30]. In this study, the material extract/indirect method was chosen over the direct method as in the latter, the cells are susceptible to trauma from abrasion or crushing. In addition, material extracts offer the advantage of measuring the dose–response relationship. A material extract can also be sterilized simply by filtration and offers the ability to evaluate its effect on cell cultures regardless of the cell proximity or lack of the material extract [31]. Concentrations of 3.125 and 6.25 mg/mL were selected for the nanoHA–silica–GIC material extract, and concentrations of 6.25 and 12.5 mg/mL were selected for the cGIC material extract in this study. This was based on a previous study where the authors evaluated the cell viability of DPSCs by treating the material extracts of nanoHA–silica–GIC and cGIC at 200, 100, 50, 25, 12.5, 6.25, and 3.125 mg/mL [32] using 3-[4,5-dimethylthiazole-2-yl]-2,5-diphenyltetrazolium bromide (MTT) assay. A previous study reported the highest cell viability percentage at concentrations of 3.125 mg/mL (96.57%) and 6.25 mg/mL (92.21%) for nanoHA–Silica–GIC and 6.25 mg/mL (92.65%) and 12.5 mg/mL (89.93%) for cGIC [33]. These concentrations were achieved by serially diluting half the original material extracts of nanoHA–silica–GIC and cGIC. This was done by transferring 2.5 mL of extract serially into a series of six 15 mL tubes that contained 2.5 mL of complete growth media. This resulted in material extracts with concentrations of 200, 100, 50, 25, 12.5, 6.25, and 3.125 mg/mL.

### 2.3. RNA Extraction

After each time interval, DPSCs were trypsinized and transferred into a 15 mL centrifuge tube. The cells were centrifuged at 12,000 rpm for 5 min. The supernatant was discarded, and each cell pellet was re-suspended using 1 mL of phosphate-buffered saline (PBS) and transferred into a 1.5 mL microcentrifuge tube and centrifuged at 13,000 rpm for 5 min. The supernatant was discarded, and the cell pellet was used for ribonucleic acid (RNA) extraction using an InnuPREP RNA Mini Kit (Analytik Jena, Jena, Germany).

### 2.4. Real-Time Reverse Transcription Polymerase Chain Reaction

A quantitative analysis of the gene expression of dentin sialophosphoprotein (*DSPP*), dentin matrix protein 1 (*DMP1*), osteocalcin (*OCN*), osteopontin (*OPN*), alkaline phosphatase (*ALP*), collagen type I (*COL1A1*), and runt-related transcription factor 2 (*RUNX2*) relevant to odontogenic differentiation of cells was performed. The extracted RNA was amplified using SensiFAST SYBR Hi-Rox One-step (Bioline, London, UK) according to the manufacturer’s instruction. The primer sequences of odontogenic marker genes were based on previous studies: *DSPP* [34], *DMP1* [34], *OCN* [35], OPN [35], *ALP* [35], *COL1A1* [34], and *RUNX2* [36]. Glyceraldehyde-3-phosphate dehydrogenase (*GAPDH*) was used as the housekeeping gene [34]. Real-time reverse transcription polymerase chain reaction (rRT–PCR) conditions were as follows: 45 °C for 10 min, 95 °C for 2 min, followed by 40 cycles of 95 °C for 5 s; 60 °C for 10 s, and 72 °C for 5 s. The experiment was carried out in triplicate. The results were analyzed using the 2^ΔΔ*C*^_T_ method [37]. In this formula, *C*_T_ values at each time point were normalized to the house keeping gene, *GAPDH*, in the same sample. Then, the *C*_T_ values were further normalized to *C*_T_ values of control samples at the corresponding time points. Briefly, the *C*_T_ values of the gene of interest (GOI) in both the experimental sample(s) and calibrator(c) (control sample) were adjusted in relation to a normalizer (norm) gene’s (endogenous control/*GAPDH*) *C*_T_ for the same two samples. The resulting 2^−ΔΔ*C*^_T_ value was incorporated to determine the fold change in expression using the equations below.

$$\Delta C_{T}\;\mathrm{sample}=C_{T}\;\mathrm{GOI}\;s-C_{T}\;\mathrm{norm}\;s$$

$$\Delta C_{T}\;\mathrm{calibrator}=C_{T}\;\mathrm{GOI}\;c-C_{T}\;\mathrm{norm}\;c$$

$$\Delta \Delta C_{T}=C_{T}\;s-C_{T}\;c$$

$$\mathrm{Fold}\;\mathrm{change}=2^{-\Delta \Delta C_{T}}$$

The results are expressed as the mean ± standard error of mean (SEM).

### 2.5. Statistical Analysis

The data were analyzed using SPSS version 20 (SPSS Inc., Chicago, IL, USA). The Kolmogorov–-Smirnov test showed that the data were normally distributed, and therefore, parametric statistical tests were performed (analysis of variance, followed by Tukey’s test; Dunnett’s test for multiple comparisons). The significance level was set at *p* < 0.05.

## 3. Results

**Figure 1:**
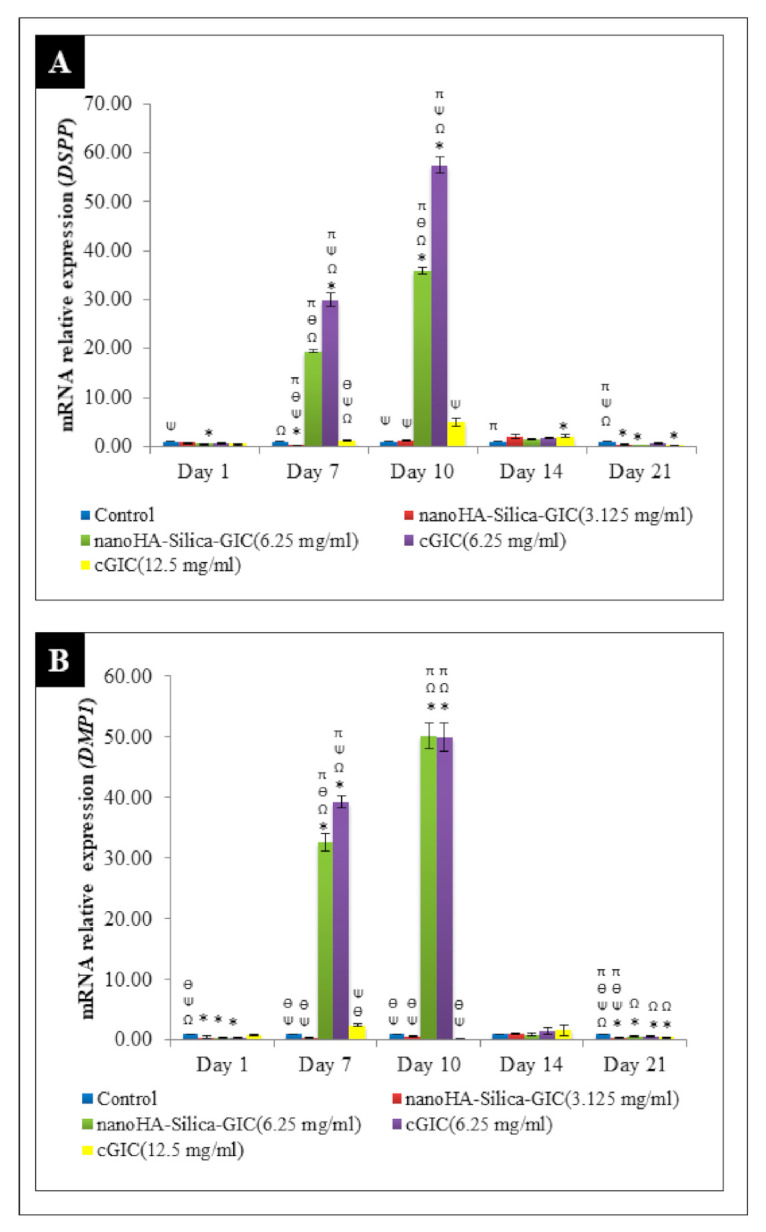
Expression of odontogenic gene markers (**A**). Dentin sialophosphoprotein (*DSPP*) and (**B**). Dentin matrix protein 1 (*DMP1*) by real-time reverse transcription polymerase chain reaction (rRT–PCR) in dental pulp stem cells (DPSCs). The data are presented as the mean ± standard error of mean (SEM). * indicates a significant difference compared to the control. Ω indicates a significant difference compared to nanohydroxyapatite-silica-glass ionomer cement (nanoHA–silica–GIC) (3.125 mg/mL). Ψ indicates a significant difference compared to nanoHA–silica–GIC (6.25 mg/mL). Ө indicates a significant difference compared to conventional glass ionomer cement (cGIC) (6.25 mg/mL). π indicates a significant difference compared to cGIC (12.5 mg/mL).

The gene expression analyses were carried out to determine the expression of odontogenic markers in DPSCs. The odontogenic markers selected for this study were *DSPP*, *DMP1*, *OCN*, *OPN*, *ALP*, *COL1A1*, and *RUNX2*. These genes were normalized with *GAPDH*. Untreated DPSCs were used as a control throughout the study. The results are expressed as mRNA relative expression. The mRNA expression of *DSPP* increased after day 1 and peaked on day 10 in all the groups (Figure 1A). However, the expression decreased after day 14 followed by day 21. Furthermore, the expression levels in the 6.25 mg/mL nanoHA–silica–GIC and cGIC were significantly higher than those in other treatment groups and control group on days 7 and 10. However, there was no significant difference between nanoHA–silica–GIC and cGIC groups on days 14 and 21. *DMP1* mRNA expression showed an increase after day 1 and peaked on day 10 in all groups (Figure 1B). However, the expression decreased after day 14 followed by day 21. On day 10, the fold change of the expression in 6.25 mg/mL nanoHA–silica–GIC and cGIC was 50.18 and 49.97, which was higher than those in 3.125 mg/mL nanoHA–silica–GIC and 12.5 mg/mL cGIC and control groups, respectively, with no significant difference between them.

**Figure 2:**
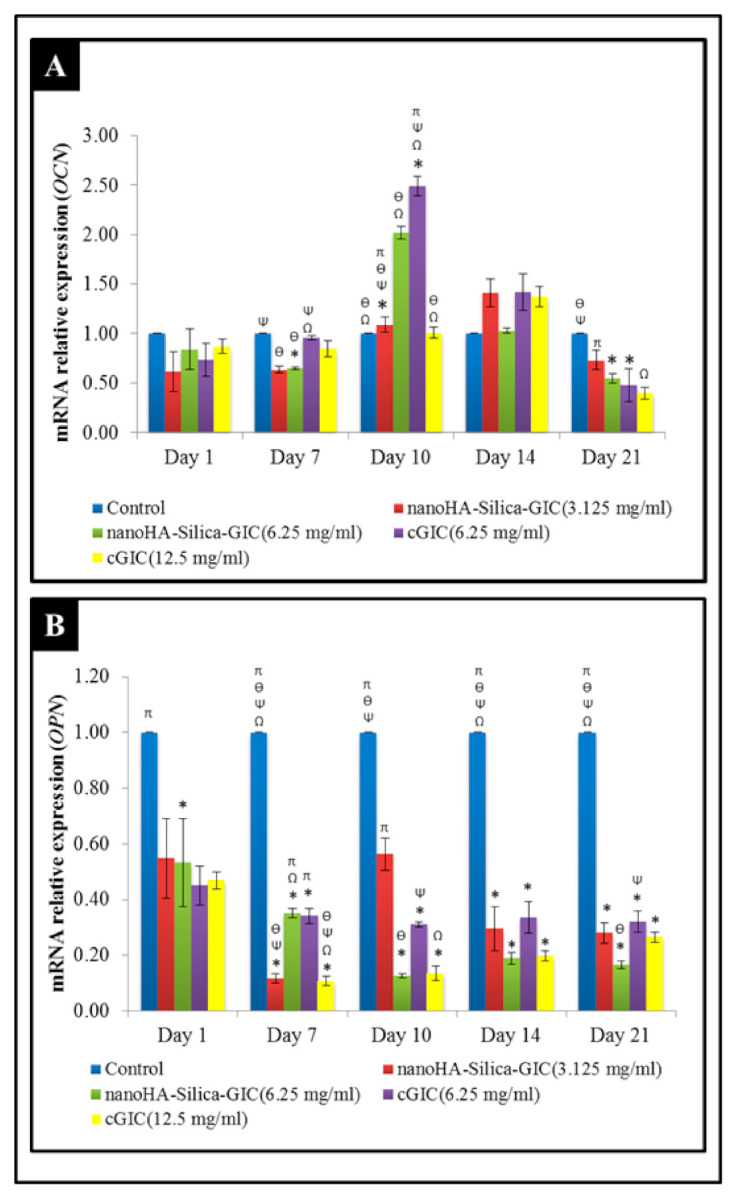
Expression of odontogenic gene markers (**A**) Osteocalcin (*OCN*) and (**B**) Osteopontin (*OPN*) based on real-time reverse transcription polymerase chain reaction (rRT–PCR) in dental pulp stem cells (DPSCs). The data are presented as the mean ± standard error of mean (SEM). * indicates a significant difference compared to the control. Ω indicates a significant difference compared to nanohydroxyapatite-silica-glass ionomer cement (nanoHA–silica–GIC) (3.125 mg/mL). Ψ indicates a significant difference compared to nanoHA–silica–GIC (6.25 mg/mL). Ө indicates a significant difference compared to conventional glass ionomer cement (cGIC) (6.25 mg/mL). π indicates a significant difference compared to cGIC (12.5 mg/mL).

*OCN* expression was not altered significantly among all groups on days 1 and 14 (Figure 2A). However, the fold change of *OCN* mRNA expression in the 6.25 mg/mL nanoHA–silica–GIC and cGIC groups was 1.09 and 2.49, which was higher than that in other treatment groups and control group, respectively, on day 10, which showed a significant difference between them. The expression of *OPN* fluctuated in all the groups as illustrated in Figure 2B. In addition, *OPN* expression in treatment groups was lower than that in the control group at all time points.

**Figure 3:**
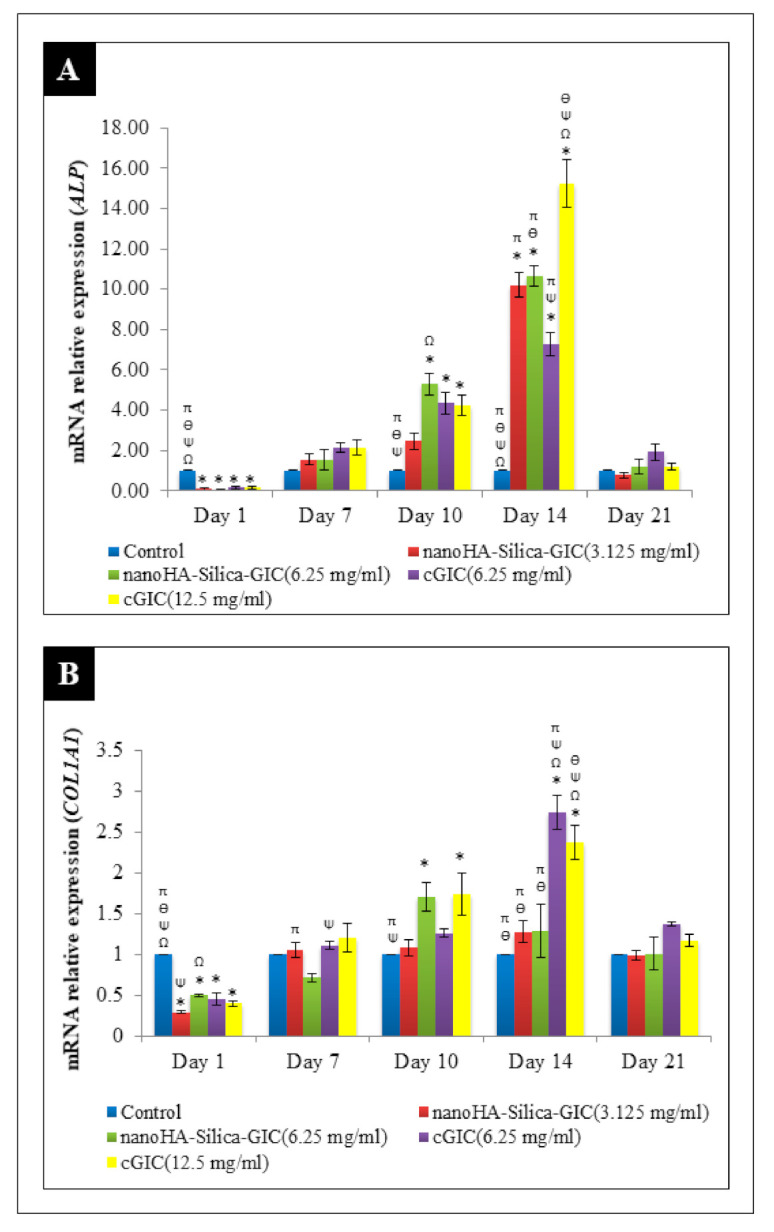
Expression of odontogenic gene markers (**A**) Alkaline phosphatase (*ALP*) and (**B**) Collagen type I (*COL1A1*) based on real time reverse transcription polymerase chain reaction (rRT–PCR) in dental pulp stem cells (DPSCs). The data are presented as the mean ± standard error of mean (SEM). * indicates a significant difference compared to C (control). Ω indicates a significant difference compared to nanohydroxyapatite-silica-glass ionomer cement (nanoHA–silica–GIC) (3.125 mg/mL). Ψ indicates a significant difference compared to nanoHA–silica–GIC (6.25 mg/mL). Ө indicates a significant difference compared to conventional glass ionomer cement (cGIC) (6.25 mg/mL). π indicates a significant difference compared to cGIC (12.5 mg/mL).

The mRNA expression of *ALP* increased on day 7 and peaked on day 14 in all the groups (Figure 3A). However, the expression of *ALP* declined after day 21. On day 14, 12.5 mg/mL cGIC had the highest fold change of 15.467 when compared with other groups. It was noted that there was significant difference between 12.5 mg/mL cGIC and other groups on day 14 (*p* < 0.05). For *COL1A1* expression, cells treated with nanoHA–silica–GIC and cGIC exhibited a time-dependent increase from day 7 to day 14 (Figure 3B). Moreover, the highest up-regulation was seen at 6.25 mg/mL cGIC compared with other treatment groups and the control group. The mRNA expression of *COL1A1* declined on day 21 where there was no significant difference between all groups (*p* > 0.05).

**Figure 4:**
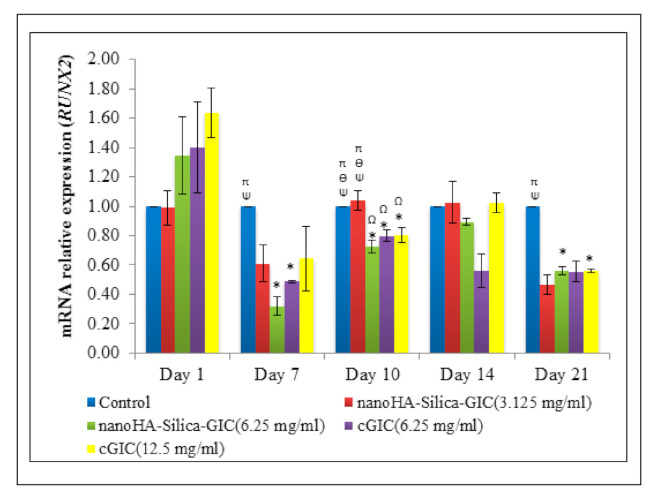
Expression of odontogenic gene marker, Runt-related transcription factor 2 (*RUNX2*) based on real time reverse transcription polymerase chain reaction (rRT–PCR) in dental pulp stem cells (DPSCs). The data are presented as the mean ± standard error of mean (SEM). * indicates a significant difference compared to C (control). Ω indicates a significant difference compared to nanohydroxyapatite-silica-glass ionomer cement (nanoHA–silica–GIC) (3.125 mg/mL). Ψ indicates a significant difference compared to nanoHA–silica–GIC (6.25 mg/mL). Ө indicates a significant difference compared to conventional glass ionomer cement (cGIC) (6.25 mg/mL). π indicates a significant difference compared to cGIC (12.5 mg/mL).

The mRNA expression of *RUNX2* increased on day 1 where the difference was not significant among all the groups (Figure 4). However, a low expression of *RUNX2* was detected in all the groups after day 7.

## 4. Discussion

GICs have wide application in clinical dentistry including as liners and bases, fissure sealants, restorative materials, and also as bonding agents for orthodontic brackets [38]. The addition of HA to GICs improves the biocompatibility of GICs and also the mechanical characteristics. Additionally, it has the ability to enhance the bond strength to the tooth structure because of its similar composition and structure to enamel and dentin [39]. In addition, Gu and colleagues reported that GICs containing 4 wt% HA particles exhibited enhanced mechanical properties in comparison with commercial GICs which could be due to the continuous formation of aluminium salt bridges, which provided the final strength of the cements [7]. NanoHA has significant remineralizing effects on initial enamel lesions [18,19]. Moshaverina and colleagues focused on the addition of nanoHA and fluorapatite (FA) to cGICs and reported that the nanoHA/FA added cements exhibited higher mechanical strength and higher bond strength to dentin as compared with the control group [18]. It was also reported that both nanoHA and FA are involved in the acid–base reaction of the GIC and react with inorganic/organic components of the GIC network through their phosphate and calcium ions. During the reaction, after H+ ions attack the ceramic particles, there would be more Ca^2+^ ions available for cement formation, polysalt bridge formation, and cross-linking, therefore reinforcing the GIC matrix [18].

Despite this, research continues to enhance the mechanical properties of GICs with the aim of expanding their indications and clinical applications. A number of markers have been well identified to be directly and indirectly involved in odontogenic differentiation. These include *DSPP*, *DMP1*, *OCN*, *ALP*, *OPN*, *COL1A1*, and *RUNX2* [34,35,36]. Therefore, these genes were investigated with *GAPDH* as the housekeeping gene in this study.

DSPP, a member of small integrin-binding ligand N-linked glycoproteins (SIBLINGs), is widely regarded as a specific marker of odontoblast. DSPP is expressed more in the dentin than in the bone and it regulates the progress of dentin formation [40]. In the current study, the gene expression level of *DSPP* was up-regulated significantly on day 7 and reached a peak on day 10 in treatment groups. This is in accordance with the fact that *DSPP* is a marker for early odontogenic differentiation as reported previously where the expression of *DSPP* was higher during primary dentinogenesis than secondary dentinogenesis in odontoblast formation [41]. This showed that DSPP functions during primary dentinogenesis and is involved in odontoblast differentiation [41]. On day 14 and 21, the expression of *DSPP* was down-regulated which may be due to the cells entering their terminal differentiation state. The expression of *DSPP* mRNA was significantly increased in the 6.25 mg/mL nanoHA–silica–GIC and cGIC groups compared with the control group and in 3.125 mg/mL nanoHA–silica–GIC and 12.5 mg/mL cGIC groups on days 7 and 10. These results indicated that nanoHA–silica–GIC and cGIC at certain concentrations may increase the expression of *DSPP*, especially during the early stages of differentiation, which contributed to the odontoblastic differentiation of DPSCs.

*DMP1* plays a regulatory role in collagen matrix organization and dentin mineralization. It is expressed during early odontoblast differentiation [25,26]. Previous studies reported that *DMP1* is expressed prior to the expression of *DSPP* and regulates DSPP gene transcription [42,43,44]. In the present study, *DMP1* expression was up-regulated from day 7 to day 10 and reached a maximum on day 10, suggesting that *DMP1* expression is necessary in the early stage of odontogenesis. However, after day 14 and day 21, the gene expression of *DMP1* was down-regulated until day 21 indicating that the cells entered terminal differentiation. Moreover, DMP1 is shown to bind specifically with the DSPP promoter during early odontogenic differentiation. It was reported that DMP1 activates DSPP transcription which explains the synchronized expression of *DSPP* and *DMP1* in the study [45]. Moreover, the expression levels in 6.25 mg/mL nanoHA–silica–GIC and cGIC on day 10 were 50.18 and 49.97 times those in 3.125 mg/mL nanoHA–silica–GIC and 12.5 mg/mL cGIC and control groups, respectively, with no significant difference. The result showed that nanoHA–silica–GIC and cGIC may promote early odontogenic differentiation.

OCN, a gamma-carboxyglutamic acid containing protein, is always expressed during the late period of odontoblast and osteoblast differentiation [46]. The expression of *OCN* showed an up-regulation on day 10, where high expression was exhibited in the 6.25 mg/mL nanoHA–silica–GIC and cGIC groups. This result indicated that nanoHA–silica–GIC and cGIC at a particular concentration might promote odontoblast differentiation. OPN is a phosphoprotein expressed in differentiating osteoblasts. *OPN* is highly expressed during the last stage of bone formation, the mineralization period [35]. The results demonstrated that *OPN* expression in treatment groups was lower than that in the control group at all times which indicated that neither nanoHA–silica–GIC nor cGIC promoted osteogenic differentiation.

*ALP* plays a crucial role in mineral deposition and is an important marker during the early stage of differentiation [47,48]. In the present study, the expression of *ALP* gradually increased and showed the highest up-regulation on day 14 followed by a decline in all groups on day 21. Similarly, it was demonstrated based on RT–PCR that the expression of *ALP* progressively increased in DPSCs after 5 and 10 days of culture [49]. However, in their study, osteogenic medium was used instead of αMEM to culture the cells. In addition, our result also showed that 12.5 mg/mL cGIC had the highest fold change of 15.467 when compared with other groups on day 14. The findings demonstrated that cGIC at a higher concentration induces early DPSC differentiation compared to cGIC at a lower concentration.

COL1A1 is the predominant collagen in dentin and constitutes the fundamental framework that supports cellular proliferation, migration, and mineralization. It is expressed by osteoblastic and odontoblastic cells at all stages during development and throughout life [50,51]. In addition, it forms a template for the controlled deposition of calcium phosphate [52]. The current findings showed that the expression of *COL1A1* was up-regulated from day 7 to day 14 indicating that odontoblast differentiation had taken place in DPSCs. The findings are consistent with the previous study that reported that COL1A1 is one of the first extracellular matrix components to be expressed [52].

RUNX2 regulates tooth and bone development in the early stages. Besides direct regulation of tooth and bone development, *RUNX2* also regulates tooth and bone development through RUNX2-related signaling pathways such as Osterix (*Osx*) [53]. Based on the current result, *RUNX2* was up-regulated on day 1 and down-regulated from day 7 onwards. The results are in agreement with the previous findings in an in vivo study which stated that *RUNX2* expression was down-regulated in the dental pulp cells and odontoblasts at the later stages of tooth development [53]. Thus, it was reported that RUNX2 was not involved in the dental pulp cell and odontoblast differentiation at the late stage. Interestingly, few studies have also demonstrated that RUNX2 can inhibit terminal differentiation of odontoblasts [54,55].

Fujita et al. in 2016 studied the effects of pre-reacted glass-ionomer (PRG) cement on the odontogenic differentiation of human dental pulp cells derived from deciduous teeth (hDPC-Ds) using alkaline phosphatase (ALP) activity and immunocytochemistry [24]. These authors reported that the PRG cement extracts significantly enhanced the ALP activity, ALP staining in the extracellular matrix of hDPC-Ds, and also the release of F and Al ions which enhanced the differentiation of hDPC-Ds. Another group of researchers investigated the odontogenic differentiation of DPSCs cultured for three weeks on hydrogel scaffolds derived from bone extracellular matrix (bECM) and compared that with those seeded on collagen I (Col-I) [25]. They evaluated the gene expression of *DSPP*, *DMP1*, and matrix extracellular phosphoglycoprotein (*MEPE*) using quantitative reverse transcription–polymerase chain reaction (qRT–PCR) and mineral deposition using Von Kossa staining. The mRNA expression levels of *DSPP*, *DMP1*, and *MEPE* were significantly upregulated with DPSCs cultured on bECM hydrogels in comparison to those cultured on Col-I scaffolds; there was more mineral deposition observed on bECM hydrogel scaffolds based on Von Kossa staining, establishing the potential of bECM hydrogel scaffolds in odontogenic differentiation of DPSCs [25]. In another study [26], the mRNA levels of *DMP1* and *DSPP* using real-time RT–PCR were analyzed to study the odontogenic differentiation of DPSCs on TCP scaffolds (three-dimensional culture) cultured for 21 days. They reported that both the genes were up-regulated and concluded that TCP possessed odontogenic-inducing potential. The results of the current research are in agreement with the previous studies [25,26] where an up-regulation was observed in the case of both *DSPP* and *DMP1*. Mohamed and colleagues studied the effect of three bioactive materials, namely, nanoHA, mineral trioxide aggregate, and calcium-enriched mixture cements, on the odontogenic differentiation of DPSCs isolated from human third molars [27]. They classified the cultured cells incubated for 14 days according to biomaterial supplementation either in odontogenic differentiation medium or in growth medium, studied the relative expressions of *Enamlysin* and *DSPP* by real-time RT–PCR, and reported that all the materials in their study promoted the odontogenic differentiation of DPSCs. Another study evaluated the effects of TEGDMA and HEMA on odontogenic differentiation of HDPCs [28]. They mimicked the clinical situations by treating the HDPCs with resin monomers for 24 h prior to analyzing the mRNA expression of genes related to pulp cell differentiation. They found that the mRNA expression of *DSPP*, *OCN*, and *OPN* was downregulated by resin monomers after a culture period of 12 days [28]. In line with that, Bakapoulou et al. also reported that deciduous teeth stem cells exposed to HEMA and TEGDMA reduced or completely inhibited the expression of markers *BSP*, *DSPP*, and *OCN* and hence the odontogenic differentiation potential leading to compromising pulp-tissue homeostasis and repair [29]. The attachment of cells to material surfaces has been shown to participate in cell proliferation, migration, and differentiation [56] for which scanning electron microscopy (SEM) has been suggested to improve visualisation through observation of cell morphology and material–cell interactions [57]. Hii et al., based on their SEM study, reported that nano-HA–silica–GIC and cGIC favored the attachment of dental pulp stem cells [33].

In conclusion, the expressions of both *DSPP* and *DMP1* were higher on days 7 and 10 and also comparable at a concentration of 6.25 mg/mL between the nanoHA–silica–GIC and cGIC groups. In the case of *OCN*, the mRNA expression in the 6.25 mg/mL nanoHA–silica–GIC and cGIC groups was higher than that in other treatment groups and the control group, respectively, on day 10, which were also comparable. However, the expression of *OPN* in treatment groups was lower than that in the control group at all time points. Despite the mRNA expression of *ALP* peaking on day 14 in all the groups, the expression was comparable between nanoHA–silica–GIC and cGIC at 6.25 mg/mL on day 10. For *COL1A1* expression, cells treated with nanoHA–silica–GIC and cGIC exhibited a time-dependent increase from day 7 to day 14 with the expression comparable between nanoHA–silica–GIC and cGIC at 6.25 mg/mL on day 10. The mRNA expression of *RUNX2* increased on day 1 where the difference was not significant in all the groups, with the expression being comparable between nanoHA–silica–GIC and cGIC at 6.25 mg/mL. The promotion effect could be due to the bioactive properties of GICs. As mentioned earlier, GICs release sodium, fluoride, silicate, and phosphate ions into surrounding aqueous media [58]. It was reported that silicate promotes osteoblast proliferation and gene expression through bone mineralization, collagen synthesis, cross-linking of the connective tissue, and metabolism [59]. The results of this study are based on an in vitro model to evaluate the odontogenic differentiation potential of the test materials which may not typically simulate the clinical situation as the material is applied to vital tissues comprising different types of cells such as ameloblasts and odontoblasts, blood, and interstitial fluids. Moreover, the response of related cell populations to the material may be affected by the placement of test materials in the oral cavity. This finding offers empirical evidence indicating that nanoHA–silica–GIC plays a role in the odontogenic differentiation of DPSCs and hence can be used as a potential restorative material in clinical dentistry.
